# Real world study of safety and efficacy of lorlatinib as second line and beyond in *ALK*-rearranged advanced non-small cell lung cancer patients in India – a multicentre chart review study (ROSELAND)

**DOI:** 10.3332/ecancer.2024.1667

**Published:** 2024-02-13

**Authors:** Bivas Biswas, Nikhil S Ghadyalpatil, Shekar Patil, Amol Patel, Sandip Ganguly, Anvesh Rathore, Bhupesh Guleria, Cpalli Firdouse Tarannum, Joydeep Ghosh, Mary Sravani Kondapally, Ravi Thippeswamy, Shashidhara Haragadde Poppa Reddy, Somnath Roy

**Affiliations:** 1 Department of Medical Oncology, Tata Medical Center, Kolkata 700160, India; 2 Department of Medical Oncology, Yashoda Hospitals, Somajiguda, Hyderabad, Telangana 500082, India; 3 Department of Medical Oncology, HGC Cancer Centre, Bangalore, Karnataka 560027, India; 4 Department of Medical Oncology, INHS Asvini, Mumbai, Maharashtra 400005, India; 5 Department of Medical Oncology, Army Hospital (R&R), Delhi 110010, India; 6 Department of Medical Oncology, Command Hospital, Pune, Maharashtra 411001, India; 7 SVS Medical College, Mahabubnagar, Telangana 509001, India

**Keywords:** real-world, safety, lorlatinib, MET, ALK

## Abstract

**Background:**

Lorlatinib, an anaplastic lymphoma kinase (ALK)–inhibitor, is approved as frontline as well as subsequent line of therapy in ALK-rearranged advanced non-small cell lung cancer (NSCLC). There is limited literature about safety and efficacy of lorlatinib in Indian patients.

**Materials and methods:**

This was a retrospective multicentre study on patients with ALK-rearranged advanced NSCLC received lorlatinib as second line and beyond between May 2017 and December 2021. ALK was tested either by immunohistochemistry or fluorescent in-situ hybridisation. Clinicopathologic features, treatment details, toxicity and outcomes were analysed.

**Results:**

A total of 38 patients were enrolled with a median age of 54 years (range: 30–72) and male: female ratio of 20:18. Fifteen (44%) patients had brain metastases at baseline. Lorlatinib use was – second line in 11 (29%), third line in 21 (55%) and fourth line in 4 (11%) of patients, respectively. The best radiologic response to lorlatinib was – complete response in 9 (24%), partial response in 17 (46%), stable disease in 9 (24%) and progressive disease in 2 (5%) of patients, respectively. After a median follow-up of 76.6 months (95% CI: 68.9–100), the median progression-free survival (PFS) of lorlatinib was not reached (95% CI: 24.3–not reached) and median overall survival (OS) of the whole cohort was 93.1 months (95% CI: 62–not reached). Both median PFS (*p* = 0.48) and median OS (*p* = 0.74) was similar between second line and later line use of lorlatinib. Thirty-three (87%) patients experienced treatment-related toxicity and six (16%) patients required dose modification.

**Conclusion:**

Lorlatinib was highly efficacious in terms of overall response rate, median PFS and median OS in this small real-world cohort of advanced *ALK*+ve NSCLC with a manageable safety profile.

## Introduction

Non-small cell lung cancer (NSCLC) is a heterogeneous group of disease and characterised by many novel molecular alterations with mutations and fusions. Anaplastic lymphoma kinase (*ALK*) rearrangements represent a unique subset, found in 5%–8% of advanced NSCLC [1–3]. The outcome of *ALK*-positive advanced NSCLC has been revolutionised with the discovery of crizotinib, a first-generation *ALK* inhibitor, and its approval in this setting in 2011 [4].

Subsequently many second-generation and third-generation *ALK* inhibitor tyrosine kinase inhibitor (TKI) got approval for use in this subgroup in different setting [5–11].

Limitation of all *ALK* TKI, like- crizotinib, ceritinib, alectinib and brigatinib was development of resistance through on-target and off-target pathway. The major limitation of crizotinib was the lack of intracranial activity. On-target mechanism was mostly through the development of secondary *ALK* mutations and the frequency of mutations increased with increasing generations of TKI [12].

Lorlatinib, a potent third-generation *ALK* inhibitor TKI, was approved as post-certinib, post-alectinib progression and post-crizotinib plus one more TKI failure after a multi-cohort phase 2 study [5]. Subsequently, lorlatinib also got approved as firstline use in advanced *ALK*-positive NSCLC after excellent results in CROWN study [13]. Lorlatinib is active against most of the documented *ALK* mutations after progression on most of the first- and second-gen TKI and has high intracranial activity [14].

Very few studies published data on real-world safety and efficacy on lorlatinib in ≥1-line use setting and those data have limitation of a small sample size with mixed results [15–21]. Only a brief report is available from India on the same setting [22]. Here, we report a multicentre retrospective cohort data on real-world safety and efficacy on lorlatinib in *ALK* rearranged advanced NSCLC from India.

## Materials and methods

### Patients

In this retrospective study, we collected data from four cancer centres in India. Patients of recurrent or metastatic NSCLC with* ALK* rearrangements who received lorlatinib second line or later line were enrolled in this study from May 2017 (lorlatinib compassionate access program started in India since February 2017) till December 2021. All patients were ≥18 years of age with at least one measurable lesion by Response Evaluation Criteria in Solid Tumours (RECIST), version 1.1 [23]. Patients who received lorlatinib through any clinical trial were excluded from this study. Ethical committee approval was obtained from respective institutions. Baseline clinicopathologic features, disease burden, previous treatment modalities, treatment-related grade-3 or 4 toxicity (according to the US National Cancer Institute Common Terminology Criteria for Adverse Events, version 4.0) [24] and outcome data were collected from hospital medical records.

### Diagnostic work-up

*ALK* rearrangement was determined by immunohistochemistry (IHC) with the Ventana method (D5F3 clone) and/or by break-apart fluorescent *in-situ* hybridisation. All patients were staged with either contrast-enhanced computed tomography (CT) of the thorax and whole abdomen with/without bone scan (when indicated) or whole-body 18-fludeoxyglucose positron emission tomography coupled with a CT scan. Magnetic resonance imaging of the central nervous system (CNS) was performed if symptomatic or as per individual institutional practice.

### Treatment

Patients received lorlatinib after at least one prior *ALK*-TKI failure. Prior cytotoxic chemotherapy use was allowed. The starting dose of lorlatinib was 100 mg once daily and was continued until clinical and/or radiological progression, development of unacceptable toxic effects or death. Lorlatinib was continued beyond RECIST progression if a patient had the clinical benefit as assessed by the treating physician. Patients with symptomatic brain metastases were treated as per multidisciplinary tumour board decision with either craniotomy and tumour excision or stereotactic radio-surgery or whole brain radiation or a combination of these modalities. Dose modification was done in case of grade 3 or grade 4 toxicity after an initial dose interruption. Two levels of lorlatinib dose modification were allowed – 75 mg followed by 50 mg. Lorlatinib response assessment was done by appropriate imaging technique as per institutional practice. After lorlatinib failure (radiological and/or clinical progression), every effort was made to do a re-biopsy followed by further molecular analysis to detect any post-lorlatinib resistance mechanism.

### Statistical analysis

Descriptive statistics were used for demographic and clinical characteristics. The Student *t*-test or Wilcoxon rank-sum test was applied for correlation between categorical and continuous variables. Chi-square or Fisher exact test was used to detect associations between qualitative variables. Survival was estimated with the Kaplan-Meier method, and survival estimates were compared using the log-rank test. Progression-free survival (PFS) for lorlatinib was calculated from the date of starting of lorlatinib to the date of disease progression. Those who died without disease progression were censored for PFS at the date of death. Data were censored on 30th April 2023. Patients who were lost to follow-up were censored at the date of last contact/follow-up. Overall survival (OS) was calculated from the date of diagnosis to the date of death from any cause. Patients who were lost to follow-up or who had abandoned treatment were also included in the PFS and OS analyses, and the outcomes for these patients were confirmed by telephone contact. STATA/SE 13.0 (StataCorp, College Station, TX, USA) was used for statistical analysis.

## Results

### Clinicopathologic feature

A total of 38 patients were identified from 4 participating centres with a median age of 54 years (range: 30–72) and male: female ratio of 20:18. Baseline details are mentioned in Table 1. Most patients had CNS imaging at the baseline line and received appropriate CNS-targeted therapy.

### Lorlatinib uses

All patients received 100 mg – once daily starting dose of lorlatinib. Lorlatinib use was – second line in 11 (29%), third line in 21 (55%) and fourth line in 4 (11%) of patients, respectively, 1 patient each received lorlatinib as fifth and sixth line of treatment. The previous lines of TKI use with sequencing are detailed in Table 2.

### Treatment outcome of lorlatinib

The best radiologic response to lorlatinib was – complete response in 9 (24%), partial response in 17 (46%), stable disease in 9 (24%) and progressive disease in 2 (5%) of patients, respectively. At the data cut-off, 14 patients were dead, 4 patients were lost to follow-up and 20 patients were alive on treatment (17 patients were on lorlatinib and 3 patients were on subsequent therapy upon progression after lorlatinib).

**Figure 1.**
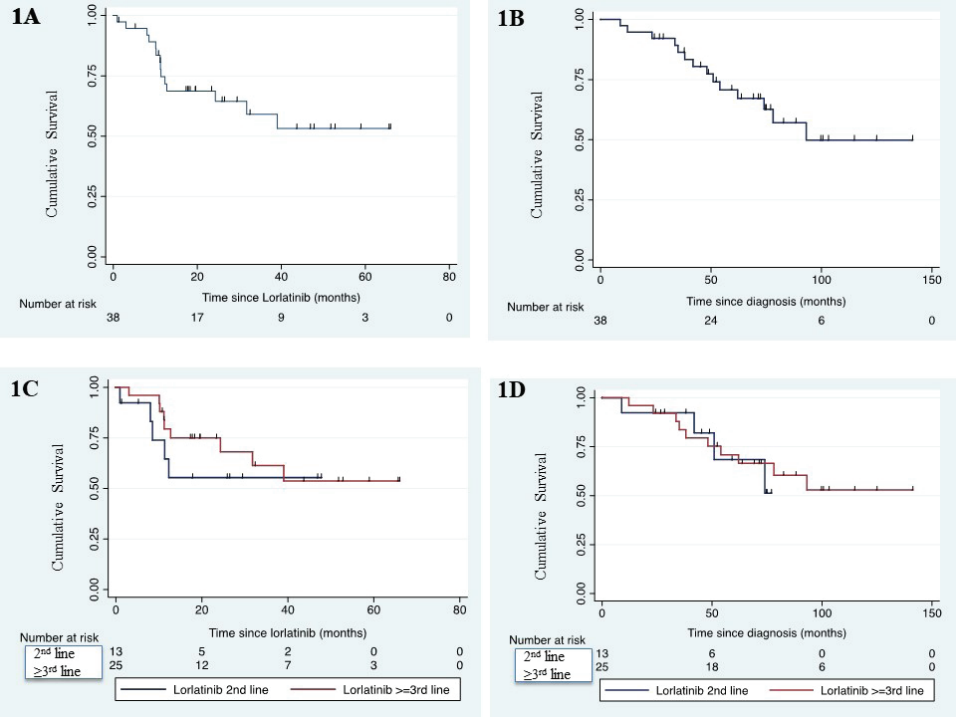
Kaplan-Meier survival curve showing median PFS in whole cohort (panel-A), median OS in whole cohort (panel-B), median PFS as per setting of lorlatinib use (panel-C) and median OS as per setting of lorlatinib use (panel-D).

After a median follow-up of 76.6 months (95% CI: 68.9–100), the median PFS of lorlatinib was not reached (95% CI: 24.3–not reached) (Figure 1A). Median OS of the whole cohort was 93.1 months (95% CI: 62–not reached) (Figure 1B). Median PFS was similar between second line or later line use of lorlatinib (HR: 0.67, *p* = 0.48) (Figure 1C). Median PFS for second line and third line lorlatinib was not reached (95% CI: 8.0–not reached) and not reached (95% CI: 12.7–not reached), respectively. Median OS was similar between second line or later line use of lorlatinib (HR: 0.82, *p* = 0.74) (Figure 1D).

### Toxicity and dose modification

Thirty-three (87%) patients experienced treatment-related toxicity (Table 4) and most toxicity were grade 1 or grade 2. Common toxicities were – oedema in 10 (26%) patients, hypercholesterolemia in 25 (66%) patients and increased blood pressure in 4 (11%) patients. Six (16%) patients required dose modification to 75 mg once daily for lorlatinib-related grade 3 or grade 4 toxicity. None of the patients had permanent discontinuation of lorlatinib due to toxicity.

### Post-lorlatinib treatment

Post-lorlatinib re-biopsy data are available for two patients and both of them had high Mesenchymal Epithelial Transition (MET) amplification. Both patients achieved partial response when treated with capmatinib. Three patients received platinum-based doublet chemotherapy and one patient received alectinib.

## Discussion

In this multi-institutional study, after a median follow-up of 76.6 month median PFS was not reached in our cohort with a median OS of 93.1 months and overall response rate (ORR) of 70%. This outcome was much high as compared to phase 2 study of lorlatinib by Solomon *et al* [5]. Our outcome was similar to another large real-world study by Peled *et al* [21] but much better than other real-world studies (Table 3). There was significant heterogeneity in the outcome results published by different real world studies. This difference in outcome is multi-factorial and can be due to different ethnic group, heterogeneous patient population, different types of TKI used, different lines of lorlatinib used, post-oligoprogression use of lorlatinib, different methods of clinical outcome monitoring, etc. In our study, majority (>80%) of lorlatinib use was after only 1–2 lines of TKI failure and very less use of alectinib and brigatinib.

Lorlatinib showed a median PFS of 5.5 months and ORR of 32% after the failure of second-generation *ALK* TKI [5]. In our study, 28 patients received at least 1 second-generation *ALK* inhibitor, either ceritinib or alectinib (Table 2). Median PFS was not reached for second or third line use of lorlatinib in our cohort.

Brain metastasis is very common in ALK+ve NSCLC, either at presentation [25] or during the course. In our study, 44% of patients had brain metastases during diagnosis and 49% brain metastases when started on lorlatinib. Lorlatinib is very effective in preventing brain metastasis [26] and also have a high intracranial response rate.

Recent phase 3 CROWN study [13, 26, 27] showed excellent tumour control with median PFS not reached at 36 months with excellent intracranial tumour control (intracranial response rate of 82%) and manageable safety profile. After this result, lorlatinib is now approved in first line setting use as the preferred first line option. Our study patients received lorlatinib as ≥ second line use before the approval of first line lorlatinib use and most of the patients received free medication through compassionate access program by Pfizer. Access to lorlatinib is a major barrier for treatment of patients with *ALK*+ve advanced NSCLC in low- and middle-income countries (LMIC). Not much therapeutic development happened so far to overcome lorlatinib resistance and subsequent disease progression. There is no standard of care in that setting other than platinum-based chemotherapy.

MET amplification is one of the merging resistances to lorlatinib [28]. We have two patients who had high MET amplification after progression on lorlatinib. Both of the patients responded to capmatinib, a potent selective MET inhibitor. There are several *ALK* inhibitors are available for treatment of patients with *ALK*+ve advanced NSCLC and they have different clinical activity, intracranial tumour control, different toxicity profiles and different resistance patterns. Optimal sequencing of these agents to get maximum survival while maintaining quality of life is a challenge. Patients in our cohort achieved a satisfactory median OS of 93.1 months.

Lorlatinib has a slightly different toxicity profile, other than class side effect of *ALK* inhibitors, like, CNS side effects, deranged lipid profile, etc. Most of the real-world studies (Table 3) reported lorlatinib-induced toxicities in patients ranging from 50% to 94% and our study reported similar frequency. Hypercholesterolemia, a classical side effects of lorlatinib [5, 13], was less in our cohort as we started statin concomitantly with lorlatinib. Two of our patients had severe neuro-cognitive dysfunction and managed conservatively with dose reduction to 75 mg/day. Toxicity profile reporting is very heterogeneous in real-world studies due to less data capture of treatment-related toxicities in out-patients and less vigorous reporting criteria. Only 16% of patients required dose modification in our cohort which was lower as compared to the landmark phase 2 study [5] and CROWN trial [13, 26, 27].

Our study results and interpretation were limited by the retrospective nature of the study which inherently carries many biases including missing data. Our study patients didn’t have any data on genomic variants of *ALK* fusion type as *ALK* V.3 variants may have better sensitivity of lorlatinib with higher efficacy [15]. None of the patients had re-biopsy upon progression on second line ceritinib or alectinib, and directly received lorlatinib as a subsequent line of treatment. Post-TKI re-biopsy access or practice is not common and does not affect subsequent treatment changes in a resource poor setting like India where access to lorlatinib and other investigation drugs is limited.

## Conclusion

In conclusion, consistent with clinical trial data and real-world studies, lorlatinib was highly efficacious in terms of ORR, median PFS and median OS in this small real-world cohort of advanced *ALK*+ve NSCLC with manageable safety profile. The outcome data were much better than many previous studies of lorlatinib after failure with first or second gen* ALK* inhibitor. There is a huge unmet need for lorlatinib in this population in resource-poor LMIC.

## Conflicts of interest

BB: Received PI grant in clinical trial from AstraZeneca, ROCHE, Pfizer, IQVIA, NOVARTIS and Johnson & Johnson and paid to Institution.

SG: Received PI grant in clinical trial from AstraZeneca, Johnson & Johnson and NOVARTIS and paid to Institution.

JG: Received PI grant in clinical trial from AstraZeneca and paid to Institution.

SR: Received PI grant in clinical trial from AstraZeneca and paid to Institution.

Rest of the authors: No conflicts of interest to declare.

## Funding

The study has been supported by an educational grant from Pfizer (Investigator-Sponsored Research Study Tracking # 67570957). This funding was solely for conduct of the study. The funding agency had no role in study design, in the collection, analysis and interpretation of data or in the decision for publication of the study.

## Informed consent

Informed consent waiver was received from Institutional Review Board (IRB) due to retrospective and non-interventional nature of the study. Respective participating centres have taken IEC/IRB approval.

## Ethical approval

The study was approved Institutional Review Board (Ref No. – 2021/TMC/234/IRB41). The study was performed in accordance with the ethical standards as laid down in the 1964 Declaration of Helsinki and its later amendments.

## Author contributions

Conceptualisation: BB, NSG, SP, AP, SG, JG and SR; formal analysis: BB, AP, SG and JG; funding acquisition: BB; project administration: BB, SG, AP and JG; supervision: BB, SG, JG and AP; data collection: all authors; writing – original draft: all authors; writing – review and editing: all authors; and approval of final draft: all authors.

## Figures and Tables

**Table 1.** Baseline demography and clinicopathological features (*n* = 38).

Variable	Number (%) Median (range)
Age (years)	54 (30–72)
Gender	
Male	20 (53%)
Female	18 (47%)
ALK detection method	
IHC	19 (50%)
Fluorescent *in-situ* hybridisation	19 (50%)
Brain metastasis (*n* = 34)	
Absent	19 (56%)
Present	15 (44%)
CNS-directed therapy (*n* = 15)	
Surgery	4 (27%)
Stereotactic radiosurgery	2 (13%)
Surgery + stereotactic radiosurgery	2 (13%)
Whole brain radiotherapy	7 (47%)
Brain metastases at the time of lorlatinib (*n* = 37)	
Absent	19 (51%)
Present	18 (49%)

*ALK*, anaplastic lyphoma kinase; CNS, central nervous system

**Table 2.** Sequencing of ALK-TKI.

	Crizotinib	Ceritinib	Alectinib	Lorlatinib	Chemotherapy*
First line	27	4	0	–	7
Second line	6	16	2	11	3
Third line	–	4	0	21	3
Fourth line	–	1	1	4	2
Later lines	_	_	_	2	2

*ALK*, anaplastic lymphoma kinase; TKI, tyrosine kinase inhibitor

* Chemotherapy regimen was platinum based- either with pemetrexed or paclitaxel

**Table 3.** Real world evidence on lorlatinib in ALK+ve advanced NSCLC.

Author	*N*	Use of lorlatinib	Prior TKI	CNS metastasis	ORR	AE	Median PFS	Median OS
Orlov *et al* [15]	35^a^	Second line – 16 Third line – 12 Fourth line – 5	Crizotinib – 17 Ceritinib – 17 Alectinib – 2	27	43%	89%	21.8 months	70.1 months
Alexander *et al* [16] (LOREALAUS)	38	Second line – 15 Third line – 13 Fourth line – 5 Later line – 5	First gen TKI – 12 Second gen TKI – 20	19	44%	60%^b^	7.3 months^c^; 13.2 months^d^	70 months
Zhu *et al* [17]	76	Second line – 7 Third line – 18 Fourth line – 19 Later line – 32	Crizotinib – 66 Ceritinib – 46 Alectinib – 43 Brigatinib – 10 Ensartinib – 1	64	33%	~70%	9.3 months	Not reached
Talreja *et al* [22]	34	Second line – 0 Third line – 18 Fourth line – 11 Later line – 5	Crizotinib – 34	Not reported	56%	94%	9.6 months	53.5 months
Lee *et al* [18]	10	Third line – 10	Crizotinib – 10 Ceritinib – 7 Alectinib – 2 Brigatinib – 1	7	67%	83%	6.5 months	Not reached
Hochmair *et al* [19]	37	Second line – 10 Third line – 23 Fourth line – 13 Fifth line – 1	Crizotinib – 25 Ceritinib – 21 Alectinib – 14 Brigatinib – 27	19	43%	49%	4.4 months^e^	41.8 months
Goto *et al* [20]	221	Second line – 154 ≥Third line – 67	Alectinib – 221 Ceritinib – 38	Not reported	Not reported	Not reported	5 months^e^	Not reported
Peled *et al* [21] (GLASS)	106	Second line – 16 Third line – 40 Fourth line – 33 ≥Fourth line – 17	Crizotinib – 40 Ceritinib – 25 Alectinib – 15 Brigatinib – 13	72	60%	46%	Not reached	89.1 months
Current study (ROSELAND)	38	Second line – 11 Third line – 21 Fourth line – 4 ≥Fifth line – 2	Crizotinib – 33 Ceritinib – 25 Alectinib – 3	15	70%	87%	Not reached	93.1 months

AE, adverse event; ALK, anaplastic lymphoma kinase; CNS, central nervous system; NSCLC, non-small cell lung cancer; ORR, overall response rate; OS, overall survival; PFS, progression free survival; TKI, tyrosine kinase inhibitor

a Two patients received lorlatinib as first line use

b 23 patients had thromboembolic events. No details about any other toxicity

c PFS in whole group

d PFS in patients who received ≥30 days of lorlatinib

e Median duration of therapy

**Table 4.** Toxicity profile.

Toxicity	Grade (*n*)	Number (%)
Hallucination	Grade 2 (1), grade 1 (1)	2 (5%)
Loose motion	Grade 2 (2)	2 (5%)
Peripheral oedema	Grade 3 (2), grade 2 (2), grade 1 (6)	10 (26%)
Increased blood pressure	Grade 2 (4)	4 (11%)
Vomiting	Grade 3 (1), grade 2 (1)	2 (5%)
Hyper cholesterolemia	Grade 3 (2), grade 2 (10), grade 1 (13)	25 (66%)
Skin rash	Grade 3 (1)	1
